# LA-ICP-MS and SIMS U-Pb and U-Th zircon geochronological data of Late Pleistocene lava domes of the Ciomadul Volcanic Dome Complex (Eastern Carpathians)

**DOI:** 10.1016/j.dib.2018.03.100

**Published:** 2018-03-27

**Authors:** Réka Lukács, Marcel Guillong, Axel K. Schmitt, Kata Molnár, Olivier Bachmann, Szabolcs Harangi

**Affiliations:** a MTA-ELTE Volcanology Research Group, 1117, Budapest Pázmány Péter sétány 1/C, Budapest, Hungary; b Institute of Geochemistry and Petrology, Department of Earth Sciences, ETH Zürich, Clausiusstrasse 25, 8092 Zürich, Switzerland; c Institute of Earth Sciences, Ruprecht-Karls University, Heidelberg, Germany; d Department of Petrology and Geochemistry, Eötvös Loránd University, 1117, Budapest Pázmány Péter sétány 1/C, Budapest, Hungary

## Abstract

This article provides laser-ablation inductively coupled plasma mass spectrometry (LA-ICP-MS) and secondary ionization mass spectrometry (SIMS) U-Pb and U-Th zircon dates for crystals separated from Late Pleistocene dacitic lava dome rocks of the Ciomadul Volcanic Dome Complex (Eastern Carpathians, Romania). The analyses were performed on unpolished zircon prism faces (termed rim analyses) and on crystal interiors exposed through mechanical grinding an polishing (interior analyses). ^206^Pb/^238^U ages are corrected for Th-disequilibrium based on published and calculated distribution coefficients for U and Th using average whole-rock and individually analyzed zircon compositions. The data presented in this article were used for the Th-disequilibrium correction of (U-Th)/He zircon geochronology data in the research article entitled “The onset of the volcanism in the Ciomadul Volcanic Dome Complex (Eastern Carpathians): eruption chronology and magma type variation” (Molnár et al., 2018) [1].

## Specifications Table

**Table:** 

Subject area	*Earth Sciences*
More specific subject area	*Geochronology, Geochemistry*
Type of data	*Tables*
How data was acquired	*Laser-ablation inductively coupled mass spectrometry (LA-ICP-MS); Thermo Element XR Sector Field (SF)-ICP-MS with Resonetics Resolution 155 laser ablation system (ETH Zürich, Switzerland) and Secondary ion mass spectrometry (SIMS; Heidelberg, Germany)*
Data format	*LA-ICP-MS: U-Th-Pb isotopic data in.xlsx format corrected for relative sensitivity, drift, alpha-dose and Th-disequilibrium*
	*SIMS: U-Th-Pb data in.xlsx format relative sensitivity-corrected and Th-disequilibrium-corrected*
Experimental factors	*Zircon grains were extracted from bulk lava rocks*
Experimental features	*Separated zircon grains were mounted in epoxy resin, polished and mapped by cathodoluminescence technique.*
Data source location	*Ciomadul Volcanic Dome Complex (Eastern Carpathians) as reported in*Table 1.
Data accessibility	*Supplementary materials*

## Value of the data

• These data provide high-spatial resolution U-Pb and U-Th zircon ages for the Late Pleistocene Ciomadul Volcanic Dome Complex (Eastern Carpathians); these ages date zircon crystallization and define a maximum age of the eruption.
• The difference between zircon crystallization and eruption ages represents the zircon residence time, which infer the time of magma residence underneath Ciomadul Volcanic Dome Complex.
• The dataset can be used to refine Th-disequilibrium corrections for (U-Th)/He zircon geochronology [1].
• Data enable recognition of redeposition, erosion, and sedimentary transport of volcanic rocks and components from the Ciomadul Volcanic Dome Complex.
• These ages are valuable for detrital zircon geochronology in the basins of the Eastern Carpathians, and can identify sediment provenance.

## 1 Data

In this article, we report in-situ U-Th and U-Pb zircon geochronological data from dacitic lava of the Ciomadul Volcanic Dome Complex (Eastern Carpathians). Data were generated using different sample preparation strategies and analytical methods: LA-ICP-MS and SIMS analyses were carried out on sectioned and polished crystal interiors, as well as on unpolished crystal surfaces (rims). LA-ICP-MS U-Pb results of more than 500 spot analyses are listed from 8 crystal populations (sample) along with 5 zircon reference materials (e.g. GJ-1, Plešovice, 91500, AUSZ7-1, AUSZ7-5 [2], [3], [4], [5], [6], [7]) performed in three sessions. 46 U-Th and 50 U-Pb SIMS results are reported, representing 4 samples (secondary references were AS3 [8] for U-Th and 91500 for U-Pb). The dataset contains the LA-ICP-MS raw and processed data and the SIMS processed data.

## 2 Experimental design, materials and methods

### 2.1 Sample collection

Localities with GPS coordinates and lithology of the samples are shown in Table 1.

**Table 1:** Details of sample localities and analyses types.

**Sample code**	**Location**	**GPS coordinates**		**Lithology**	**Analyses type**
		**N**	**E**		
CSO-NM1	Murgul Mare - *Nagy Murgó*	46°4'36"	25°48'01"	andesite	LA-ICP-MS interior
CSO-PD2	Pilişca - *Piliske*	46° 9'16"	25°50'47"	dacite	LA-ICP-MS interior
CSO-MB-M	Malnaş quarry *- Málnás*	46°2'59"	25°48'43"	shoshonite	LA-ICP-MS interior
CSO-MB-B	Bixad quarry - *Bükszád*	46°5'1"	25°50'1"	shoshonite	LA-ICP-MS interior
CSO-BL1	Baba-Lapoşa - *Bába-Laposa*	46°10'33"	25°52'27"	dacite	LA-ICP-MS interior
CSO-NH5	Dealul Mare - *Nagy-Hegyes*	46°6'06"	25°55'9"	andesite	LA-ICP-MS interior
CSO-BH	Puturosul - Büdös-hegy	46°7'11"	25°56'55"	dacite	LA-ICP-MS interior, SIMS rim
					
CSO-BAL03	Cetatea Balvanyos - *Bálványos*	46°6'54"	25°58'4"	dacite	LA-ICP-MS rim
CSO-BAL(cs)					SIMS rim
CSO-AB	Turnul Apor - *Apor-bástya*	46°8'6"	25°51'51"	dacite	SIMS rim
CSO-KHM1	Haramul Mic - *Kis-Haram*	46°10'38"	25°55'25"	dacite	SIMS rim, interior

### 2.2 Sample preparation

Zircon crystals were separated from the 63–125 µm size fraction of rock samples by standard gravity and magnetic separation methods.

For LA-ICP-MS analyses the separated zircon grains, except for the crystals of CSO-BAL03, were mounted in 1 in. epoxy resin mount and polished to a 1 µm finish. Before dating, zircons were checked by optical microscopic and cathodoluminescence (CL) imaging. CL imaging was produced using an AMRAY 1830 SEM equipped with GATAN MiniCL and 3 nA, 10 kV setup at the Department of Petrology and Geochemistry, Eötvös University, Hungary. Crystals of sample CSO-BAL03 were mounted in 1 in. epoxy resin mount and measured without polishing. For SIMS measurements zircon grains were mounted in epoxy and in indium, details of these preparatory works are presented in Table 2.

**Table 2:** Analytical background for SIMS U-Pb and U-Th analysis performed at University of Heidelberg.

Mounting type	Epoxy and Indium
Sample preparation and treatment before SIMS analysis	**Work procedure***(for Epoxy Mounts)*
	1. Ground down & polished with SiC paper (FEPA# 800, 1200, 2400, 4000) & diamond paste (1 µm, 1/4 µm) 2. Cleaned with distilled water & methanol (before CL imaging at SEM) 3. Carbon-coated (Quorum Q150T ES); thickness of carbon coating: 18 nm 4. CL imaged at SEM 5. Carbon-coating removed by polishing 6. Cleaned with EDTA + NH_3_, distilled water & methanol (before SIMS analysis) 7. Gold-coated (Quorum Q150T ES); Thickness of gold coating: 50 nm
	**Work procedure***(for Indium Mounts)*
	1. Standard imbedded, ground down & polished with SiC paper (FEPA# 800, 1200, 2400, 4000) & diamond paste (1 µm, ¼ µm). 2. Samples imbedded by pressing crystals into indium metal 3. Cleaned with EDTA + NH_3_, distilled water & methanol (before SIMS analysis) 4. Gold-coated (Quorum Q150T ES); Thickness of gold coating: 50 nm

Age calibration approach	U-Th, U-Pb
Analytical conditions	U-Th conditions are described in [9]; U-Pb conditions in [10]
	Beam diameter: U-Th ~ 40 µm (Köhler Ap.: 400 µm), U-Pb ~ 20 µm (Köhler Ap.: 200 µm)
	Primary beam intensity: U-Th ~ 40 nA, U-Pb ~ 17 nA
	Mass resolution (M/DM): ~ 4500
	Pre-raster conditions: U-Th 15 µm, 10 s, U-Pb 15 µm, 30 s

Software to calculate ages	ZIPS 3.1.1
Method to calculate ages	U-Th: two-point isochron using zircon and melt with Th/U = 3.58 [11], U-Th RSF = 1.095 ± 0.020
	U-Pb: ^207^Pb-corrected ^206^Pb/^238^U ages, disequilibrium corrected using melt with Th/U = 3.58 [11] and using constant *D* value = 0.3354 + 0.0632 [12]
Primordial lead model	Surface contamination ^207^Pb/^206^Pb = 0.8469
	
Standard	AS3 (U-Pb calibration; 1099.1 Ma [8]), UO_2_/UO vs. Pb/U, *n* = 27, rel. uncertainty = 2.4%
	91500 (U concentration [5]), U/^94^Zr_2_O RSF = 0.00424 (*n* = 1)
	
Secondary standard	U-Th: AS3 (^230^Th)/(^238^U) = 0.990 ± 0.006 (MSWD = 0.69, *n* = 33)
	U-Pb: 91500 = 1055 ± 27 Ma (*n* = 1)

Comments	^230^Th half-life [13], all other half-lives [14]

### 2.3 LA-ICP-MS and SIMS analyses

LA.ICP-MS analyses were performed at the Department of Earth Sciences, ETH Zürich, Switzerland, and the SIMS analyses at the HIP Lab of the Institute of Geosciences, Heidelberg University, Germany. Analytical setups are presented in Table 2, Table 3.

**Table 3:** Analytical background for LA-ICP-MS U-Pb analysis performed at ETH Zürich.

**Laboratory name**	**Department of Earth Sciences, ETH Zürich**
**Laser ablation system**	
Make, Model & type	ASI Resolution 155
Ablation cell & volume	Laurin Technics 155, constant geometry, aerosol dispersion volume < 1 cm^3^
Laser wavelength	193 nm
Pulse width	25 ns
Fluence	~ 2 J cm^−^^2^
Repetition rate	5 Hz (4 Hz in session 160413)
Spot size	30 µm (40 µm in session 160413)
Ablation rate	~ 75 nm pulse^−1^
Sampling mode/pattern	Single hole drilling, 5 cleaning pulses
Carrier gas	100% He
Ablation duration	40 s (25 s in session 160413)
Cell carrier gas flow	0.7 l/min
**ICP-MS Instrument**	
Make, Model & type	Thermo Element XR SF-ICP-MS
Sample introduction	Ablation aerosol only, squid aerosol homogenization device
RF power	1500 W daily tuned
Make-up gas flow	~ 1.05 l/min Ar (gas mixed to He carrier inside ablation cell funnel) daily tuned
Detection system	Single detector triple mode SEM, analogue, Faraday
Masses measured	27, 29, 31, 49, 88, 89, 91, 93, 138, 139, 140, 141, 146, 147, 153, 157, 159, 163, 165, 166, 169, 172, 175, 178, 202, 204, 206, 207, 208, 232, 235, 238 amu
Integration time per peak	5 ms (masses 27, 29, 31, 88, 89, 91, 93, 138, 139, 140), 10 ms (masses 141, 146, 147, 153, 157, 159, 163, 165, 166, 169, 172, 175, 178, 202, 208, 232, 235), 20 ms (204, 238), 25 ms (49), 100 ms (masses 206, 207)
Total integration time per reading	0.683 s (0.45 s in session 160413)
Dead time	16 ns (10 ns in session 160413)
Typical oxide rate (ThO/Th)	0.18%
Typical doubly charged rate (Ba^++^/Ba^+^)	3.5%
**Data Processing**	
Gas blank	30 s prior to each ablation spot (20 s in session 160413)
Calibration strategy	GJ-1 (used as primary calibration material in all sessions.
	Validation reference materials used in sessions:
	session 160412: Plešovice, 91500, AUSZ7-1, AUSZ7-5
	session 160413: Plešovice, 91500, AUSZ7-1, AUSZ7-5
	session 160627: Plešovice, 91500, AUSZ7-1, AUSZ7-5
	References:
	Plešovice [3], [4], 91500 [4], [5], AUSZ7-1 [6] and AUSZ7-5 [7]
Reference Material info	GJ-1 ^206^Pb/^238^U 0.09761 ± 0.0002 (weighted mean of ID-TIMS analysis ± 2*σ*, Jackson et al. [2])
Data processing package used	IOLITE v2.5, v3.4 [15], [16] with VizualAge [17]
Mass discrimination	Mass bias correction for all ratios normalized to calibration reference material
Common Pb correction	No common-Pb correction applied
Uncertainty level & propagation	Ages are quoted at 2 SE absolute, propagation is by quadratic addition. Reproducibility of reference material uncertainty (i.e. external uncertainty) is propagated.
Data handling	Validation reference materials were used to correct for alpha dose-dependent age offsets [18]. Correction was accomplished by modelling the dependence of age offset on total radiation dose, calculated from sample age and concentrations of U and Th [19] in each session. Th disequilibrium correction was performed after alpha dose-correction using the algorithm of [20], assuming a constant Th/U partition coefficient ratio of 0.33 ± 0.063 (1*σ*) [12].
