# Supplementary material for: LA-ICP-MS and SIMS U-Pb and U-Th zircon geochronological data of Late Pleistocene lava domes of the Ciomadul Volcanic Dome Complex (Eastern Carpathians)
Source: Data Brief. 2018 Mar 27;18:808–13. doi: 10.1016/j.dib.2018.03.100 (PMC5996732; doi:10.1016/j.dib.2018.03.100)
Supplement: Supplementary file 1 — Supplementary material [file mmc1.docx]

**DECLARATION**

We wish to confirm that there are no known conflicts of interest associated with this publication and there has been no significant financial support for this work that could have influenced its outcome.

We confirm that the manuscript has been read and approved by all named authors and that there are no other persons who satisfied the criteria for authorship but are not listed. We further confirm that the order of authors listed in the manuscript has been approved by all of us.

We confirm that we have given due consideration to the protection of intellectual property associated with this work and that there are no impediments to publication, including the timing of publication, with respect to intellectual property. In so doing we confirm that we have followed the regulations of our institutions concerning intellectual property.

Réka Lukács, PhD

MTA-ELTE Volcanology Research Group

Budapest, Hungary

e-mail: reka.harangi@gmail.com

on behalf of the authors
